# Rheumatoid Factor and Its Interference with Cytokine Measurements: Problems and Solutions

**DOI:** 10.1155/2011/741071

**Published:** 2011-06-22

**Authors:** Else Marie Bartels, Inger Falbe Wätjen, Eva Littrup Andersen, Bente Danneskiold-Samsøe, Henning Bliddal, Søren Ribel-Madsen

**Affiliations:** ^1^The Parker Institute, Frederiksberg Hospital, 2000 Frederiksberg, Denmark; ^2^Faculty of Health Science, University of Copenhagen, 2200 Copenhagen N, Denmark; ^3^Centre for Sensory-Motor Interaction, Aalborg University, 9220 Aalborg, Denmark

## Abstract

Use of cytokines as biomarkers for disease is getting more widespread. Cytokines are conveniently determined by immunoassay, but interference from present antibodies is known to cause problems. In rheumatoid arthritis (RA), interference of rheumatoid factor (RF) may be problematic. RF covers a group of autoantibodies from immunoglobulin subclasses and is present in 65–80% of RA patients. Partly removal of RF is possible by precipitation. This study aims at determining the effects of presence of RF in blood and synovial fluid on cytokine measurements in samples from RA patients and finding possible solutions for recognized problems. IL-1**β**, IL-4, IL-6, and IL-8 were determined with multiplex immunoassays (MIA) in samples from RA patients prior to and after polyethylene glycol (PEG 6000) precipitation. Presence of RF does interfere with MIA. PEG 6000 precipitation abolishes this RF interference. We recommend PEG precipitation for all immunoassay measurements of plasma samples from RA patients.

## 1. Introduction

Several autoantibodies are described in plasma and synovial fluid from rheumatoid arthritis (RA) patients [1]. Two of these, rheumatoid factor (RF) and anticitrullinated protein antibodies (anti-CCP), are used in routine diagnostic work. RF was first described by Waaler around 1940 [2] and is not a clearly defined molecule but a combination of auto-antibodies of the types IgM, IgG, IgA, IgD, or IgE [1, 3–5]. RFs are defined as Ig autoantibodies which bind via variable sequences of their Fab region to the Fc region of an IgG [3, 6]. RFs show high reactivity with the IgG type 1, 2, and 4, but less with type 3, indicating that the antigen specificity of IgG is not relevant, but the type of Fc region is [6, 7]. It is usually IgM and/or IgG, which are the most abundant, and IgM concentration alone, or the concentration of both together, is expressed as the RF concentration when measured in a clinical setting [8]. RF is found in plasma from 65 to 80% of subjects with RA and some other connective tissue diseases [4, 9, 10], as well as in a condition like pneumonia [11], and RF interference is therefore a very real problem when measuring samples from RA patients. IgM RF in RA is mainly of the soluble pentameric form [3, 4, 6] which shows high affinity to IgG binding.

Apart from RF and anti-CCP, there are autoantibodies which react with antigens from other species, but with weak avidity. Human antimouse antibody (HAMA), mainly IgG, is the most prevalent [12]. “Heterophilic antibodies”, among these RF, are well-known sources of interference in immunoassay of proteins in samples from human plasma, cerebrospinal fluid, or synovial fluid [13–18], but interference from most autoantibodies can easily be counteracted for, while IgM in its pentameric form is particularly reactive due to its polyvalence [12]. 

Interference from heterophilic antibodies may occur in all designs of immunoassays, but the immunometric type is known to be most exposed to this [19]. This is due to the heterophilic antibodies' ability to bridge between the capture and the detection antibodies in the immunometric setup, and thereby create a false signal.

Since multiplex immunoassay (MIA) and two-site enzyme-linked immunosorbent assay (ELISA), the most commonly applied methods for quantitative determination of cytokines, are both immunometric and mainly using detection antibodies of the IgG type, interference from RF must be considered to be a potential serious problem when measuring samples from RA patients. 

With the growing understanding of the immunological processes involved in RA, determination of cytokine profiles as biomarkers is getting more widespread and has been proposed for early detection of RA [20], differential diagnosing [21, 22], and monitoring of effect of treatment with cytokine-binding antibody preparations [15]. As a precaution against part of the heterophilic antibodies, common practice is to add various cocktails of mouse, calf, goat, and rabbit serum to assay diluents for immunoassay. The major problem left is then interference of RF in RF-positive patient samples with a high concentration of RF [4, 9, 23]. A suggested solution is to precipitate at least part of RF with polyethylene glycol 6000 (PEG 6000) [24–26]. There is a good reason for this choice, since the main suspected culprit in the interference of RFs with the IgG antibodies is believed to be the polyclonal IgM RF, and PEG 6000 will precipitate these larger complexes but very little of the lower molecular monomers [25, 27, 28]. Another suggested precipitation agent is protein L [24], but price in relation to binding capacity makes use of protein L impossible for clinical tests. An attempt to remove RF with a block copolymer of ethylene and propylene (HeterBlock) and/or protein-L has also been made [20], but the results were variable and inconclusive. 

In the present study, our aim was to determine to which degree presence and concentration of RF will interfere with MIA cytokine measurements carried out on plasma or synovial fluid from patients representing a range of RF concentrations and to validate sample pretreatment procedures using PEG 6000 precipitation to reduce possible interference from RF.

## 2. Materials and Methods

### 2.1. Patients and Samples

Samples from ten patients from the Rheumatology Clinic at Frederiksberg Hospital, age range 35 to 75 years, three men and seven women, all having active RA and fulfilling the ACR-criteria [29], were included. Five plasma samples were chosen, representing a range of RF concentrations from 8 to 1141 IU/mL, and five synovial fluid samples were chosen, representing a range of RF concentrations from 2 to 189 IU/mL. The patients were part of a group treated with cytokine-binding antibody preparations. To be classified as RF-positive means having a plasma RF concentration above a set defined value, for example, in Denmark 17 IU/mL, hence one of the plasma samples used for the study was from a patient considered RF-negative. 

All patients gave informed consent to participate in the study, which was approved by the Frederiksberg and Copenhagen Municipalities' Ethics Committee (no. KF01-256496).

Blood was drawn into K_2_EDTA-sprayed Vacutainers (BD Diagnostics, Franklin Lakes, NJ, USA) and stored directly at −80°C. Joint fluid was drawn with a syringe from the knee joint, immediately transferred to a polypropylene vial, centrifuged at RCF 500 ×g, and then the supernatant was stored at −80°C.

### 2.2. Determination of RF

The concentration of RF in the samples of plasma and synovial fluid, prior to and following PEG 6000 precipitation, was measured by immunoturbidimetrics at the Department of Clinical Biochemistry, Frederiksberg Hospital. The method used gives the RF concentration as the concentration of IgM. A nonprecipitated and the PEG 6000-precipitated aliquot of each plasma or synovial fluid sample was measured on the same day.

### 2.3. Removal of Antibodies by Precipitation with Polyethylene Glycol 6000 (PEG 6000)

Prior to measurements, samples were thawed at room temperature and mixed. An 800 *μ*L aliquot of the synovial fluid was centrifuged at RCF 16000 ×g for 30 minutes, which is our procedure for removal of ectosomes. 

Synovial fluid was treated with hyaluronidase from bovine testes (Sigma, St. Louis, Mo, USA) 5000, that is, per mL sample, incubated at room temperature for 10 minutes, in order to reduce the viscosity. 

RFs were removed from samples by PEG precipitation as described by De Jager et al. [24]. The method is effective and at the same time gentle to the cytokine analytes: a 30% solution of PEG 6000 (Merck, Darmstadt, Germany) in 0.1 M sodium phosphate buffer, pH 7.4, was added in volume ratio 1 : 10, to give a final concentration of 3% PEG 6000. Samples were mixed and left at 4°C for 60 minutes, centrifuged at RCF 700 ×g for 45 minutes, and the supernatant was used for analysis.

### 2.4. Immunoassay Reagents

The possible effect of RF on immunoassays of cytokines was evaluated by analysis in the following assays, which we have developed and validated in ELISA and MIA format: interleukins (IL-) IL-1*β* CptAb monoclonal, clone 508A 7G8 mouse IgG_1_, and 508A 4A2 mouse IgG_1_, DtcAb monoclonal, clone 508A 3H12 mouse IgG_1_, CP recombinant human, all from BS; IL-4 CptAb, DtcAb, CP recombinant human, all from BS; IL-6 CptAb monoclonal, clone 677B 6A2 mouse IgG_1_, DtcAb monoclonal, clone 505E 23C7 mouse IgG_1_, CP recombinant human, all from BS; IL-8 (CXCL8) CptAb monoclonal, clone 893A 6G8 mouse IgG_1_, DtcAb monoclonal, clone 790A 28G2 mouse IgG_1_, CP recombinant human, all from BS (“BS” is Biosource, Invitrogen Life Technologies, Carlsbad, Calif, USA).

The above-mentioned capture antibodies were covalently coupled to fluorescent, carboxylated microspheres (Luminex, Austin, Tex, USA) by the carbodiimide method advised in literature [30, 31]. The concentrations of capture antibodies in the coupling step had been titrated in numerous experiments, to result in a uniform, high density of capture antibody coupled to all the microspheres in the suspension, to make a sample of these appear as a narrow peak in flow cytometry after reaction with RPE-conjugated goat antimouse IgG.

### 2.5. Multiplex Immunoassay Procedure

The assay procedure had been optimized as to the concentrations of detection antibodies and the details of incubation times, number of washes, and assay buffer composition, resulting in following regimen: the capture antibody microspheres were blocked and stored in a phosphate buffered saline pH 7.40 (PBS) with 1% bovine serum albumin and 0.05% sodium azide. The density of the suspension of capture beads was adjusted to 1400 beads for each analyte per 25 *μ*L, and this volume was pipetted into each well of the microtitre plate. This amount of beads with capture antibody coupled to their surface had a binding capacity in far excess of the amount of analyte able to specifically bind to the capture antibody concerned. Samples of plasma or synovial fluid, in some cases PEG-precipitated, were diluted 1 : 4 with an assay diluent composed of PBS with 0.5% bovine serum albumin, and 25 *μ*L of this diluted sample were pipetted onto the inner wall of each of duplicate wells. The microtitre plates were covered with adherent foil, centrifuged at RCF 200 ×g for 1 minute to combine sample and capture bead suspension, and incubated for 90 minutes with agitation at ambient temperature in the dark. Then 25 *μ*L of the solution of the detection antibodies for all the analytes, dissolved in PBS with 0.5% bovine serum albumin, were added, the plates centrifuged at RCF 200 ×g for 1 minute, incubated for 120 minutes with agitation, and placed in 4°C refrigerator over the night. The beads were washed with 200 *μ*L PBS with agitation for 5 minutes, centrifugation at RCF 400 ×g for 6 minutes, and aspiration of the supernatant by means of a microtitre plate washer. A diluted solution of streptavidin-RPE (Molecular Probes, Eugene, Ore, USA) of 20 *μ*g/mL in PBS with 1% BSA of 20 *μ*L was added to each well, the plates gently centrifuged, incubated for 30 minutes with agitation at ambient temperature in the dark, the beads washed with 200 *μ*L sheath solution for flow cytometry (BD Biosciences, San Jose, Calif, USA), centrifuged at RCF 400 ×g for 6 minutes, and the supernatant aspirated. Finally 120 *μ*L sheath solution were added, the beads were suspended by agitation and analyzed in a FACSArray Bioanalyzer flow cytometer (Becton Dickinson Immunocytometry Systems, San José, Calif, USA).

### 2.6. Cytokine Spiking of Samples

The plasma or synovial fluid samples were spiked with the studied cytokines and added to the assay diluent. This diluent was used for dilution of samples. The resulting “Addition” samples were a neat sample, with no cytokines added, and diluted samples with a composition corresponding to a 1 : 4 dilution of an authentic sample with concentrations 3.4 pg/mL to 2500 pg/mL of each cytokine, and prepared to give 3-fold steps. In one experiment, the spiked diluent was added either prior to or following PEG precipitation, in order to evaluate the extent of cytokine loss by coprecipitation from the supernatant layer.

### 2.7. Data Handling

Bead clusters were separated in far-red versus red diagrams, gated, and median fluorescence intensity (MFI) of the reporter in yellow channel recorded. The data were calculated in the FACS Array and FCAP Array software.

## 3. Results

### 3.1. Samples

Table 1 shows the concentrations of RF of IgM type in the plasma samples P1–P5 prior to and following precipitation with PEG 6000. In Denmark, the definition of being RF-positive is to have a plasma RF concentration higher than 17 IU/mL. Hence in our material P1 corresponded to RF negative, and samples P2–P5 to RF-positive. PEG-precipitation removed around half of the RF of the IgM type in plasma. 

Table 2 shows the RF IgM concentrations in the synovial fluid samples SF1–SF5 prior to and following PEG 6000 precipitation, which removed between all and two thirds of the RF of IgM type in this type of sample material. The concentration of RF in nonprecipitated synovial fluid in proportion to the RF concentration in plasma from the same patient at the same time varied significantly. SF1 came from an RF-negative patient while, SF2–SF5 came from RF-positive patients.

### 3.2. Interference from RF

**Figure 1:**
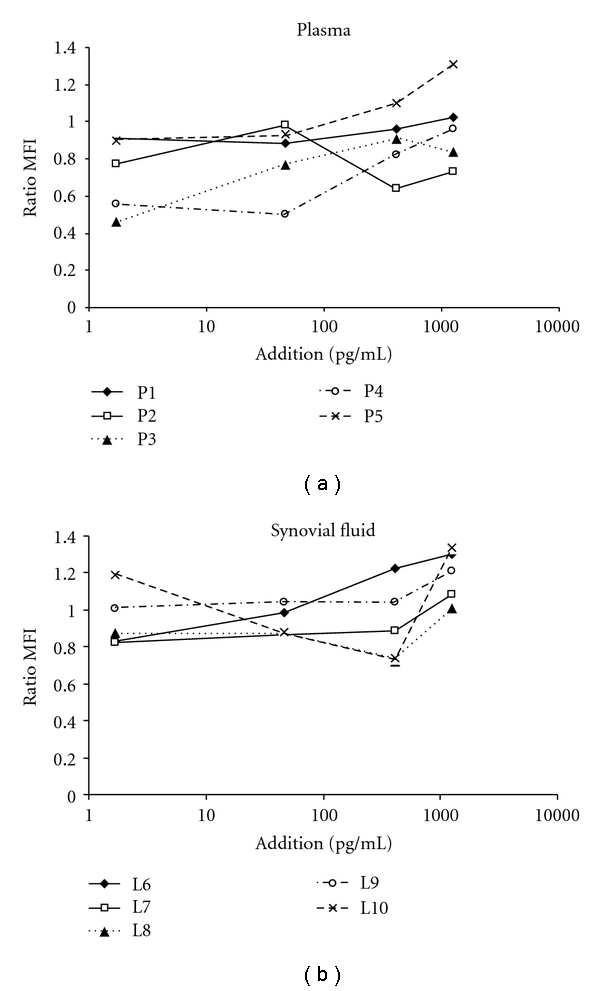
Measured concentrations in PEG 6000-precipitated and in nonprecipitated samples at different spiked concentrations of IL-1*β* for plasma (a) and synovial fluid (b).

Precipitation of RF in plasma or synovial fluid did change the interaction of a panel of cytokines with the antibody reagents used in MIA. This is illustrated by the ratio between the median fluorescence intensities (MFIs), measured by the flow cytometer, in the analysis of the PEG-precipitated and the nonprecipitated aliquot of the same sample, shown in Figures 1(a) and 1(b) for IL-1*β* in plasma and synovial fluid, respectively. IL-1*β* was chosen here due to no present baseline concentration in our samples. Notice that in P5 who had an extreme concentration of RF, little effect of precipitation of RF was seen, but the RF concentration was here still two to three times the RF concentration considered high.  Table 3 shows the concentration measured prior to and following PEG 6000 precipitation in plasma for the patient with low RF (P1) and a patient with high (P4) concentration in the region most commonly found, as well as the concentration measured prior to and following PEG 6000 precipitation in SF for the patient with low RF (SF1) and a patient with high RF (SF4) concentration. PEG precipitation does not have a great effect on SF.

### 3.3. Influence of PEG Precipitation on Cytokine Concentration

Loss of sample contents from cytokines by the PEG-precipitation was evaluated by comparison of the results from two procedures: (1) samples were spiked with the cytokines prior to precipitation and then assayed; (2) samples were precipitated, then spiked and assayed. This was done for the four cytokines IL-1*β*, IL-4, and IL-6. The results are shown in Table 4 giving the ratio between concentrations measured by method 1 and by method 2 for precipitation. No significant difference between the cytokine results from the two procedures was seen for any of the analytes. The ratio between the two values was in all cases close to unity, indicating that PEG precipitation does not precipitate cytokines and therefore does not change the true cytokine concentrations in plasma and that the apparently high cytokine concentration seen with RF-positive patients must be due to interference from RF.

### 3.4. Interference from Multiplexing

To assure that the interference of RF seen in MIA measurements was not due to the MIA setup, nonprecipitated plasma samples were assayed by multiplex and by singleplex (only capture beads and DtcAb for one analyte in the same microtiter plate well) for a selection of cytokines, IL-1*β*, IL-4, and IL-6. There was no significant difference in the measured concentrations in the two setups.

## 4. Discussion

In the present study, we have shown that RF in the concentrations which frequently occur in the plasma of RA patients [32, 33] (and own data from clinic), and probably also in a large part of synovial fluid samples from RA patients, interferes with MIA cytokine concentration measurements of plasma and synovial fluid samples from RF-positive patients. In general, the interference is likely to result in a higher artefact value than the true one. However, we do see only a minor effect on the cytokine immunoassay in a plasma sample with a very high RF concentration. This may be caused by RF binding to itself [4] due to the high distribution of RF in this particular sample, or it may simply be due to the fact that only about 50% of RF is precipitated out, leaving an RF concentration of two to three times what is usually considered a high RF. 

Interference of RF with the capture antibodies used in immunoassays could be eliminated by truncating the Fc-portion of them, but such modified capture antibodies for the range of cytokines we wanted to determine are not available on the market at present. The seen interference of RF in MIA can to a high degree be abolished by precipitation with PEG 6000. This is in accordance with earlier observations by De Jager's group [24]. The chosen precipitation method and concentration of PEG 6000 have the advantage of being gentle to the analytes in question, showing no precipitation of the cytokines when abolishing a high percentage of the RF. Since RF is a mixture of autoantibodies [1, 3, 4], one must conclude that following precipitation the remaining part of RF must be mainly the monomeric less reactive types [3, 4] which cause less interference than the precipitated part, since spiking with cytokine prior to precipitation shows the same cytokine concentration as spiking following precipitation. This is in accordance with findings of Digeon et al. [28]. The interference of RF could be an amplified effect in MIA, although it is more likely to be an effect of RF, especially caused by the IgM part's polyvalence [10]. Our results clearly support this idea, since we do not see any significant difference between singleplex and multiplex measurements. One may ask if the amount of IgG1 detection antibody for the particular cytokine would affect the influence of presence of RF on the measurements. For the cytokines in question in this study, there is a difference between amount of the different IgG1s in the assay, but there is no difference in interference seen which indicates that the setups are properly optimized. This is further confirmed by our recovery which is in the same range of the manufacturers'. The recovery does though vary to some extent with concentration, and interference at the lower concentrations will therefore give a larger problem. Overall, with PEG 6000 precipitation and a further standard correction for interference of HAMAs with addition of mouse serum and foetal bovine serum, a reliable and sensitive assay for cytokine measurements of RA samples is therefore at hand. When measuring the cytokines in SF, the interference of RF was seen to be neglectable. This is probably due to the high concentration of hyaluronic acid in SF. Hyaluronic acid is shown to initiate formation of IgG-IgM RF complexes [34] and may in this way abolish the interference.

## 5. Conclusion

In conclusion, we recommend PEG 6000 precipitation of all plasma from RA patients aimed at immunometric cytokine determination like MIA and ELISA to avoid interference from RF which otherwise may give apparently higher cytokine concentrations than the true one. PEG precipitation does not affect the cytokines present in plasma.

## Figures and Tables

**Table 1:** RF concentrations in the plasma samples prior to and following precipitation in patients 1–5 (P1–5). P1 is RF-negative, the rest are RF-positive.

Plasma sample	RFs prior to precipitation IU/mL	RFs following precipitation IU/mL
P1	4	5
P2	25	12
P3	195	85
P4	236	118
P5	1141	631

**Table 2:** RF concentrations in synovial fluid prior to and following precipitation in patients SF1–SF5. SF1 was RF-negative, SF2–SF5 were RF-positive.

Synovial fluid sample	RFs prior to precipitation IU/mL	RFs following precipitation IU/mL	RFs in plasma from the same patient IU/mL
SF1	2	1	7
SF2	34	1	85
SF3	29	13	78
SF4	54	15	54
SF5	189	60	400

**Table 3:** Cytokine concentration in nonprecipitated and PEG-precipitated plasma samples with low RF (P1) and high RF (P4) and synovial fluid samples with low RF (SF1) and high RF (SF4) as well as sensitivity for the assay and ratio between concentrations in nonprecipitated and precipitated samples for IL-1*β*, IL-4, IL-6, and IL-8 (Spiked with 417 pg/mL. Recovery consistent and as given by manufacturers).

		Concentration pg/mL nonprecipitated	Concentration pg/mL precipitated	Ratio precipitated : nonprecipitated	Baseline concentration nonprecipitated/precipitated pg/mL
IL-1*β* sensitivity: 10.5 pg/mL	P1	369	272	0,737	0/0
	P4	290,5	220	0,757	1.5/0
	SF1	169	219	1,296	0/0
	SF4	259	270	1,042	0/0

IL-4 sensitivity: 27 pg/mL	P1	334	271	0,811	0/0
	P4	309,8	245	0,791	0.2/0
	SF1	336	292	0,869	0/0
	SF4	337,6	355	1,052	3.4/0

IL-6 sensitivity: 11.5 pg/mL	P1	368	290	0,788	0/0
	P4	477,4	290	0,607	2.6/0
	SF1	293	289	0,986	43/58
	SF4	362	386	1,066	96/115

IL-8 sensitivity: 14.0 pg/mL	P1	280	225	0,804	0/0
	P4	355,8	275	0,773	9.2/0
	SF1	306	296	0,967	54/29
	SF4	387	398	1,028	88/42

**Table 4:** Ratio of cytokine concentrations measured from samples spiked with cytokine and then PEG-precipitated and samples which were PEG-precipitated and then spiked with cytokine. Mean ± SD given.

Analyte	Ratio spiked sample precipitated : sample matrix precipitated, then spiked mean ± SD
IL-1*β*	0.94 ± 0.15
IL-4	1.01 ± 0.14
IL-6	0.95 ± 0.16
